# *In Vitro* Recombination Catalyzed by Bacterial Class 1 Integron Integrase IntI1 Involves Cooperative Binding and Specific Oligomeric Intermediates

**DOI:** 10.1371/journal.pone.0005228

**Published:** 2009-04-21

**Authors:** Véronique Dubois, Carole Debreyer, Claudine Quentin, Vincent Parissi

**Affiliations:** Laboratory of Cellular and Molecular Microbiology and Pathogenicity (MCMP), UMR 5097-CNRS, University Victor Segalen Bordeaux 2, Bordeaux, France; Baylor College of Medicine, United States of America

## Abstract

Gene transfer via bacterial integrons is a major pathway for facilitating the spread of antibiotic resistance genes across bacteria. Recently the mechanism underlying the recombination catalyzed by class 1 integron recombinase (IntI1) between *att*C and *att*I1 was highlighted demonstrating the involvement of a single-stranded intermediary on the *att*C site. However, the process allowing the generation of this single-stranded substrate has not been determined, nor have the active IntI1•DNA complexes been identified. Using the *in vitro* strand transfer assay and a crosslink strategy we previously described we demonstrated that the single-stranded *att*C sequences could be generated in the absence of other bacterial proteins in addition to IntI. This suggests a possible role for this protein in stabilizing and/or generating this structure. The mechanism of folding of the active IntI•DNA complexes was further analyzed and we show here that it involves a cooperative binding of the protein to each recombination site and the emergence of different oligomeric species specific for each DNA substrate. These findings provide further insight into the recombination reaction catalyzed by IntI1.

## Introduction

The prevalence of antibiotic resistance is mainly due to the horizontal transfer of antibiotic-resistance genes, conveyed by mobile genetic elements such as plasmids and transposons. One way for the spread of antibiotic resistance-encoding genes involves integrons. Integrons are a class of site-specific recombination elements which insert and excise mobile antibiotic resistance gene cassettes, and which are located on plasmids and/or transposons. All class 1 integrons consist of two conserved sequences (CS) flanking a variable central region encompassing antibiotic resistance gene cassettes [1]. The highly conserved 5′CS includes an *intI1* gene encoding an integrase, an adjacent recombination site *att*I1 and a promoter region, while the 3′CS is more variable. Several classes of integrons have been identified according to the type of integrase, the most prevalent class 1 integrons are characterized by an *intI1* gene encoding an integrase of 337 amino acids.

Gene cassettes can exist in two forms: either as free covalently closed supercoiled circular molecules unable to replicate, or as linear molecules integrated at the *att*I1 site of integrons [2]. Gene cassettes consist of a single coding sequence carrying at its 3′ end a blaGES-1/qaxE *att*C recombination site (referred here as *att*C). The *att*C sites, also called 59-base elements, are essentially formed from two simple sites at the boundaries of an imperfect palindromic region with a 7 bp core site GTTRRRY, whilst the *att*I1 can vary in sequence and it devoid of a palindromic sequence [3]–[5]. Thus, the *att*C site appears more complex than *att*I1 and consists of two potential core sites R″-L″ and L′-R′ separated by a region that is variable in sequence and length. A number of these have been demonstrated to be efficiently recombined by IntI1 [Bibr pone.0005228-Stokes1] [for a functional and structural analysis of the site see ref 6 and 7].

The IntI1 integrase belongs to the tyrosine recombinase family and catalyzes site-specific recombination between *att*I1 and *att*C sites with site preferences. Until recently, the reaction catalyzed by the IntI1 integrase encoded by class 1 integrons has essentially been studied *in vivo*. In bacteria, IntI1 can catalyze recombination between either two *att*C, one *att*I1 and one *att*C, or two *att*I1 sites [8]. The most biologically relevant events appear the integration and excision reactions involving *att*I1×*att*I1 or *att*C×*att*C sites. Structural studies and *in vivo* recombination models demonstrated that IntI1 recombines the single bottom strand of the *att*C sites (bs*att*C), folded in an imperfect hairpin structure with extrahelical bases, and the double strand (ds) *att*I1 substrate [9], [10]. The involvement of a single strand of the *att*C suggests that the Holliday Junction (HJ) intermediate resulting from the recombination event cannot be resolved by a second strand exchange that would lead to the linearization of the replicon. Bouvier and co-workers proposed that this step could be carried out by the DNA replication process [10]. Another question remains obscure concerning the formation of the single-stranded *att*C. Several candidate mechanisms have been proposed for the generation of the ss*att*C substrate such as DNA replication, natural genetic transformation or events occurring during conjugal transfer [9]. However assessing the molecular mechanism underlying recombination was heavily constrained by the limitations of performing *in vitro* assays. That the reason why we recently focus our work on setting up an *in vitro* strand transfer assay allowing us to reproduce the initial strand exchange catalyzed by a recombinant purified IntI1 between *att*C and *att*I1 sites [11]. This system allowed us to demonstrate that recombination activity could be detected when using a ds*att*C, even if this process was strongly increased when the bs*att*C was provided. This result strongly suggests that the ss*att*C intermediate could be generated during the recombination process by the IntI1 protein in the absence of other cellular factors. However, previous DNA binding studies indicated that IntI1 has low affinity for the double-stranded *att*C compared to the bs*att*C and ds*att*I1 [12]–[14]. Taken together these data led us to study in further detail the intI1•DNA complexes involved in the recombination mechanism in order to reconcile the low affinity for the ds*att*C and the possible *in vitro* generation by Int1 of a single-strand intermediate. Using a recombinant highly purified IntI1 obtained from a new class 1 integron isolated in our laboratory in *P. aeruginosa* [15], recombination assays, crosslink analyses and gel shift experiments led us to demonstrate that i) the recombination involving the *att*C site required a cooperative binding of the enzyme to the second partner *att*I1; and ii) the quaternary structure of InIt1 involving the tetramerization of the enzyme on the corresponding *dsatt*C seems to be crucial for the generation of the ss*att*C substrate.

## Results

### *In vitro* intermolecular strand transfer between double-stranded *att*I1 and *att*C sites catalyzed by the recombinant IntI1 enzyme is inhibited by endonuclease S1

**Figure 1:**
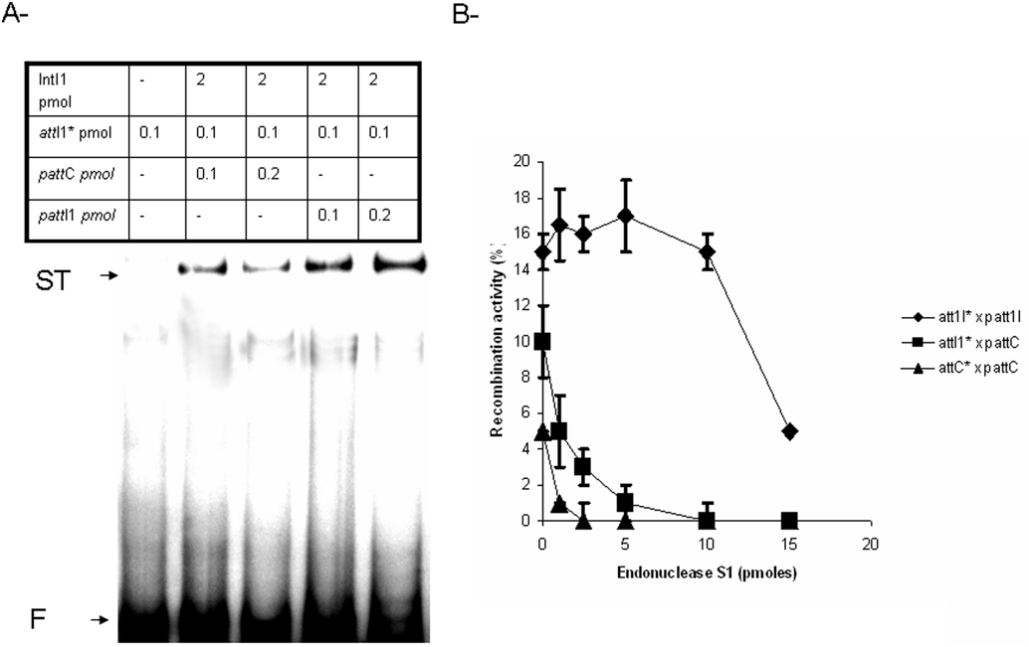
A- *In vitro* strand transfer catalyzed by IntI1 at *att*I1 and *att*C sites. Reactions were performed for 90 min in the presence of purified IntI1 (2 pmoles), 0.1 to 0.2 pmoles of either pGEM-T-*att*I1 or pGEM-T-*att*C (p*att*I1 and p*att*C in the figure) and 0.1 pmoles free 5′ ^32^P radiolabeled *att*I1 sites under the standard conditions described in materials and methods. Products were loaded on 1% agarose gel run at 200 V, for 2 hours at room temperature and autoradiographied. F: free recombination sites, ST: strand transfer products. B- Inhibition of *in vitro* strand transfer activity of IntI1 by nuclease S1. Strand transfer reactions were carried out as described in A but with increasing amounts of S1 nuclease (0 to 15 pmoles). Strand transfer products were quantified using DNAJ software and are plotted in the figure as percentage of the initial substrate. Results are the mean±standard deviation (error bars) of three independent experiments.

Recombinant pure IntI1(His)_6_ (referred as IntI1 for convenience) was tested using the recombination assay previously set up [11]. Data shown in fig 1A indicate that the reaction efficiency depends upon the recombination sites involved as previously reported [11]. Furthermore, recombination products can be clearly detected even in reactions involving an ds*att*C substrate. Previous studies demonstrated that such recombination *in vivo* requires the bottom strand of the *att*C [10]. Taken together these results suggest strongly that the ss*att*C intermediate can be generated *in vitro* in absence of other bacterial protein in addition to IntI1.

To better ascertain this, we tested the effect of nuclease S1 on the *in vitro* recombination activity of IntI1. As shown in fig 1B, the addition of nuclease S1 during the reaction induced a strong inhibition of all activity involving the *att*C sites, while it had only a slight effect on the recombination between *att*I1 substrates. No degradation of the ds*att* fragments was detected in a control experiment and no degradation of the recombination products was observed when treatment with the nuclease was performed after completion of the reaction (data not shown). This indicates that the decrease of the reaction products signal was due to an inhibition of the strand transfer reaction by the nuclease S1. Thus the increased sensitivity to S1 treatment of the *att*C recombination indicates that in the reaction involving such a substrate, an important single-stranded structure is accessible to the nuclease. This strongly suggests that the ss*att*C is generated during the reaction time in presence of IntI1.

### Cooperative binding of intI1 to *att*I1*/att*C sites

**Figure 2:**
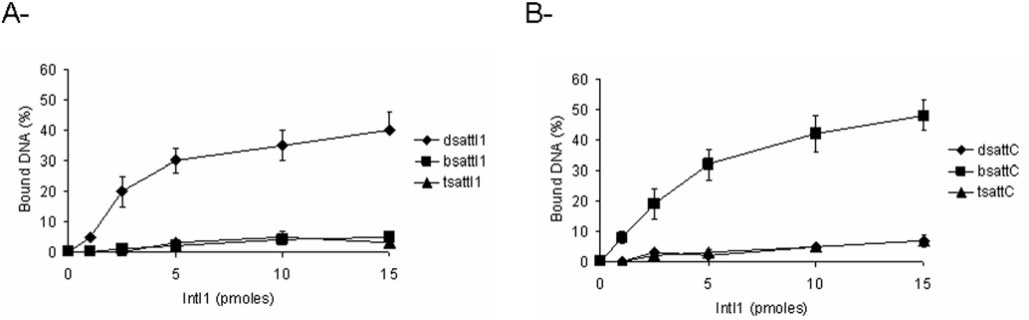
*In vitro* DNA binding of IntI1 with double- or single-stranded *att*I1 (A) and *att*C (B) sites. Free 5′ ^32^P radiolabeled dsDNA fragments containing recombination sites (0.1 pmoles) were incubated with purified IntI1 (1–15 pmoles) at 4°C for 20 min before electrophoresis on 1% agarose gel run at 50 V, for 2 hours at 4°C. Gel shifted bands were then quantified using DNAJ software and are plotted in the figure as percentage of bound DNA. Results are the mean±standard deviation (error bars) of three independent experiments.

Recombination activity catalyzed by IntI1 between the *att*I1 and *att*C sites requires correct binding of the protein on each substrate. However, differences in affinity were previously reported depending on the recombination sequence bound [11], [13], [14]. Gel shift assays performed with our recombinant protein showed that this enzyme shares the same DNA affinity reported previously. Indeed as shown in fig 2, IntI1 had a better affinity for the ds*att*I1 and the single-stranded bs*att*C while this enzyme only poorly bound the ds*att*C site.

Interestingly, IntI1 presented a very low affinity for the ds*att*C in the concentration range where ds*att*I1×ds*att*C recombination activity was detected (see fig 1), suggesting that under the reaction conditions, which differed from the binding assay conditions only by the presence of the second ds*att*I1 fragment, IntI1 can form active complexes on the ds*att*C. This could be due to a cooperative binding between the enzyme and its double-stranded substrates.

**Figure 3:**
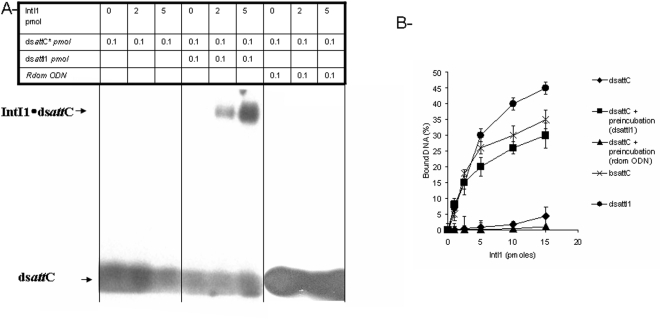
Cooperative binding of IntI1 to *att*I1*/att*C sites. A- *In vitro* DNA binding of IntI1 with double-stranded *att*C fragment in presence or not of *att*I1 site. Free 5′ ^32^P radiolabeled dsDNA *attC* fragments containing recombination sites (0.1 pmoles) were incubated at 4°C for 20 min with purified IntI1 (0–5 pmoles) after or without preincubation with ds*att*I1 (0.1 pmole) or random ODN (0.1 pmole) at 4°C for 20 min. Products were then loaded on 1% agarose gel and electrophoresis was run at 50 V, for 2 hours at 4°C. B- Effect of preincubation with different *att* fragments on *in vitro* DNA binding of IntI1. Free 5′ ^32^P radiolabeled dsDNA fragments containing recombination sites ds*att*C, ds*att*I1, or bs*att*C (0.1 pmoles) were incubated at 4°C for 20 min with purified IntI1 (0–5 pmoles) after or without preincubation with ds*att*I1 (0.1 pmole) or with a random ODN (0.1 pmole) at 4°C for 20 min. DNA binding was measured by quantification of gel shifted bands using DNAJ software and also filter binging assay as described in materials and methods section. The percentage of bound DNA was then plotted in the graphic B. Results are the mean±standard deviation (error bars) of three independent experiments.

To ascertain this hypothesis we tested the affinity of IntI1 for ds*att*C in the presence of the second partner ds*att*I1. As shown in fig 3, while IntI1 has a low affinity for the ds*att*C site in the absence of the ds*att*I1 fragment, the addition of the latter oligonucleotide (ODN) strongly increased the binding of IntI1 to the ds*att*C. In these conditions, the affinity of IntI1 for the ds*att*C fragment almost reached the level observed for the bs*att*C. No stimulation effect was observed in the presence of a random control ODN (fig 3A and B). In addition, the preincubation of the protein with ds*att*I1 did not change its affinity for the ts*att*C nor the bs*att*C (data not shown). Taken together these results support the cooperative binding of the enzyme to ds*att*I1 and ds*att*C, thus explaining the recombination activity observed between these two substrates despite the different affinities we observed.

### Characterization of the IntI1•DNA complexes fold on *att*I1 and *att*C

In view of the different behaviors of IntI1 on *att*I1 and *att*C recombination fragments, we decided to further characterize the complex fold on each site. Owing to the low stability of the complexes between IntI1 and *att*C, only poor shifts were observed in EMSA experiments using recombinant enzyme and *att*C fragment [11], [13], [14].

In order to better stabilize the complex fold on this recombination substrate, we used a previously described method to covalently block the protein•DNA complexes. This method is based on the use of a chemical crosslinking agent, *cis*-aquahydroxydiamino platinum (AHDAP). It allows the formation of covalent bonds between platinum and the potential acceptors on proteins and nucleic acids, mainly via the sulfur-containing groups of cysteine or methionine residues, the imidazole rings in histidine residues, and the N group of guanine, adenine, and cytosine [16]. We have previously described crosslinking experiments between HIV-1 IN and its viral DNA substrate with the goal of isolating the retroviral integration intermediates [17].

**Figure 4:**
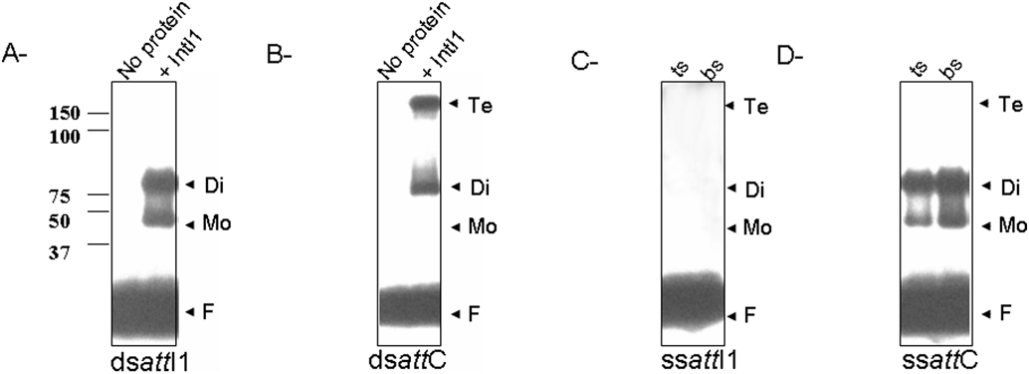
SDS-PAGE analysis of crosslinked IntI1•DNA complexes. IN (2 pmoles) was preincubated with the 5′-end radiolabeled ds*att*I1 (A), ds*att*C (B), ss*att*I1 (C, bs and ts), ss*attC* (D, bs and ts) for 30 min in the presence of AHDAP at 37°C (final volume 20 µl). Products were separated by electrophoresis on 12% SDS-PAGE gel. The gel was then dried and autoradiographed. The positions of monomers, dimers and tetramers bound to DNA were determined by comparison with the migration distance of protein weight markers (BIO-RAD) submitted to electrophoresis under the same conditions. F: free substrate.

IntI1 was treated with AHDAP in the presence of each 5′ radiolabeled ds*att* fragment. The products were then subjected to DNase I treatment in order to eliminate non-protected DNA tails and to distinguish between monomers and higher oligomeric forms of IN bound to the oligonucleotide. The stabilized complexes were analyzed on SDS-PAGE and autoradiography and the nature of the complexes was determined by comparison with the migration of known weight markers. As shown in fig 4, different complexes were detected on ds*att*C and ds*att*I1, suggesting that the binding of the enzyme on each fragment involves different specific oligomers.

Evaluation of the distance of migration of each bands in comparison with the weight marker allowed us to determine that monomers and dimers of IntI1 fold on the ds*att*I1 fragment (fig 4A) while dimers and tetramers fold on the ds*att*C fragment (fig 4B).

To better understand the formation of each complex, we further analyzed the IntI1•DNA complexes using the single-stranded *att* sites. As shown in fig 4C, no complex was isolated in the presence of both ts*att*I1 and bs*att*I1. In contrast monomers and dimers were found to bind to both single strands of *att*C (fig 4D).

Taken together these results demonstrate that specific oligomeric complexes are folded on each *att* site involving the formation of dimers and tetramers on the ds*att*I1 and ds*att*C sites respectively. Tetramers folding may result from two isolated dimers bound to each strand of the *att*C site.

## Discussion

The beneficial aspects of the introduction of antibiotics in the treatment of human infectious diseases is hampered by the emergence of bacterial resistance has become an ever-increasing problem that threatens the clinical usefulness of these drugs. The spread of antibiotic resistance genes especially via integrons is an important problem to overcome to avoid lateral large-scale diffusion of resistance. Thus the determination of integron behavior at the molecular level remains a crucial issue. Recently interesting information on the mechanism underlying the recombination catalyzed by IntI1 recombinase in bacterial class 1 integrons was described both at the structural and physiological levels [9], [10]. These data demonstrate the involvement of a single-stranded intermediate on the *att*C site during *att*C×*att*I1 recombination. The model proposed by Bouvier and co-workers suggests strongly that this single-stranded intermediate is created during DNA replication, conjugal transfer or transformation.

We previously set up an *in vitro* assay allowing to reproduce the strand transfer reaction occurring during recombination between all the *att* sites using a pure recombinant enzyme [11]. Those results suggested strongly that the single-stranded *att*C intermediate could be generated in absence of DNA replication system and in the presence of IntI1. Nuclease S1 treatment during the recombination reaction allowed us to show that the degradation of single-stranded DNA strongly inhibited all the reactions involving the *att*C site (see fig 1B). We previously showed that *in vitro* recombination was promoted when the single-stranded bs*att*C was used [11]. Taken together these results indicate that IntI1 possesses all the determinants required for the *att*C recombination reaction and that the bs*attC* intermediate can be generated during the reaction time in absence of other protein in addition to the recombinase.

*att*C recombination and formation of *bsatt*C required an initial binding of the enzyme to the double-stranded site. However, all the previous studies reported a very poor interaction of the protein for this substrate [12]–[14] see fig 2. Such low level of binding could be compensated by the cooperative binding of IntI1 to both *att*I1 and *att*C. DNA binding experiments performed in the presence of both sites allowed us to demonstrate that the binding of IntI1 for ds*attC* was greatly improved when the enzyme was preincubated with ds*att*I1 fragment in contrast to random control ODN (see fig 3). This indicates that during *att*I1×*att*C recombination, the interaction of the enzyme with the initial ds*att*C can be affected and promoted by its binding to ds*att*I1.

To better characterize the IntI1•DNA complexes involved in the reaction, we stabilized all reaction intermediates by cross-linking with AHDAP [17]. Experiments performed in the presence of each ds*att* fragment led to the detection of clearly distinct complexes (see fig 4). Indeed the binding of IntI1 to ds*att*I1 led to the formation of monomer•DNA and dimer•DNA complexes, while tetramer•DNA complexes were detected in addition to dimer•DNA on ds*att*C. Detection of dimer•DNA complexes on each strand of the *att*C fragment (see fig 4D) in contrast to *att*I1 (4C) strongly suggests that IntI1 binds both top and bottom *att*C strands to form the tetramer intermediate.

The exact mechanism for ss*att*C folding remains unclear. Helicase and topoisomerase assays performed using the pure recombinant IntI1 showed no effect (data not shown), suggesting that the generation of the ss intermediate by the recombinase itself is unlikely. Preliminary experiments indicate that the ss*att*C structure can be fold spontaneously in reaction conditions (data not shown). IntI1 could participate in the stabilization of this ss intermediate. This process could be promoted by the presence of extra-helical bases in the *att*C structure [9]. The determination of specific oligomeric complexes folded on each recombination site, and especially the detection of dimers on each strand of the *att*C resulting in the formation of a tetrameric complex bound to the ds*att*C substrate, led us to propose its involvement in the mechanism. The importance of this oligomeric intermediate is supported by a recent report of an IntI1 mutant with improved recombination rate and which is only slightly affected in the binding to the recombination sites [18]. This indicates that binding of the enzyme to its substrate is not the unique determinant for an efficient recombination but that proficient synaptic complex formation, mediated by the correct folding and oligomerization of the protein on the substrates, could be the limiting step of the process.

**Figure 5:**
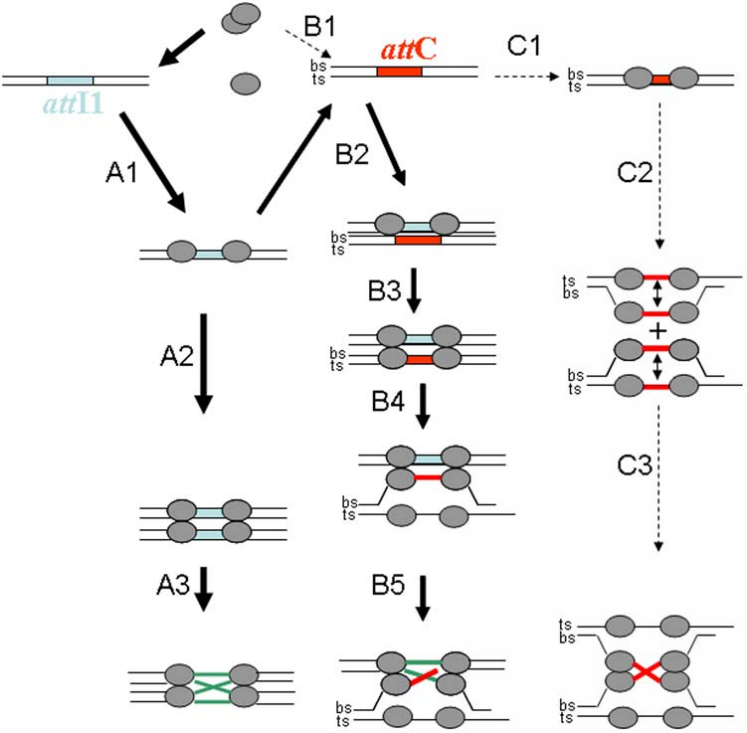
Model for the cooperative binding and the *inti1* oligomers involved in the *in vitro* recombination catalyzed by IntI1. In the *att*I1×*att*I1 recombination reaction IntI1 binds both ds*att*I1 fragment as a dimer and catalyzes the strand exchange and the formation of the HJ intermediate (lanes A1 to A3). In the case of *att*I1×*att*C recombination, IntI1 binds ds*att*C only slightly (B1). Interaction with the first *att*I1 substrate led to cooperative binding to the second *att*C site (B2), allowing the recruitment of a second IntI1 dimer on the second strand of the *att*C site (B3) and the formation of the tetrameric intermediate, leading in turn to the stabilization of a ss*att*C intermediate (B4). The strand exchange between ds*att*I1 and bs*att*C can then be catalyzed (B5). Recombination activity detected in presence of two *att*C sites suggests that the initial low binding of the enzyme to this site is sufficient for triggering all the subsequent recombination steps (way C). ts: top strand, bs: bottom strand.

Taken together, our data in addition to previous reports allow us to propose a molecular model for the *in vitro* recombination catalyzed by IntI1 (fig 5). In the *att*I1×*att*I1 recombination reaction, IntI1 binds both ds*att*I1 fragments under dimeric form and catalyzes the usual strand exchange and the formation of the HJ intermediate (way A1 to A3). In the case of *att*I1×*att*C recombination, IntI1 binds ds*att*C only slightly (B1). Interaction with the first *att*I1 substrate leads to cooperative binding to the second *att*C site (B2), thus allowing the recruitment of a second IntI1 dimer on the second strand of the *att*C site (B3) and the formation of the tetrameric intermediate, which in turn stabilizes a ss*att*C structure (B4) leading to the catalysis of the strand exchange between ds*att*I1 and bs*att*C (B5). Recombination activity detected in the presence of two *att*C sites suggests that the initial low binding of the enzyme to the site is sufficient to trigger all the subsequent recombination steps (way C). It is important to point out that the work reported here leading to this model accounts only for the recombination activity the cooperative binding and the oligomerization state of the protein on each substrate detected *in vitro*. The precise structure of each intermediate remains to be elucidated, especially the tetrameric complex formed with the ds*att*C site. In addition, the process underlying the generation of the *ssatt*C fragment also remains to be established and is under study in the laboratory.

A cellular mechanism such as DNA replication might also constitute a second pathway for generating the intermediate substrate of the recombination, in addition of being required for the final HJ resolution step. Furthermore all cellular processes favoring the umpairing of DNA as DNA replication and conjugation should promote the recombination. However, our data indicate that IntI1 possesses all the intrinsic properties for the initial strand transfer between recombination site and thus can actively participate in all the early steps of the process.

## Materials and Methods

### DNA, bacterial strains and culture media

The DNA sequence encoding the entire class I integron was previously cloned from *P. aeruginosa* Pa695 (see references 11 and 15 into pC23 vector. The *E. coli* DH5α strain was used for propagation of plasmids and *E. coli* BL21 strain was used for expression of IntI1(his)_6_ recombinant enzyme. All DNA vectors and PCR products were purified using the DNA purification systems from PROMEGA (Wizard plus SV miniprep and Wizard SV Gel kits). PCR amplifications were done under standard conditions using *Taq* polymerase (PROMEGA). Sequencing was performed by polymerase chain reaction-based sequencing (ABI Prism big dye terminator cycle sequencing ready reaction kit, Applied Biosystems).

### Cloning, expression and purification of IntI1

IntI1(His)_6_ was expressed and purified as described previously [11]. The recombinant pure enzyme IntI1(His)_6_ was referred as IntI1 in the manuscript for convenience.

### *In vitro* strand transfer assays

The *in vitro* strand transfer assay previously set up [11] was used. The ds *att*I1 containing donor substrate (100 bp) was generated by annealing the 5′^32^P radiolabeled oligonucleotide AttI1 (5′-ACGCCGTGGGTCGATGTTTGATGTTATGGAGCAGCAACGATGTTACGCAGCAGGGCAGTCGCCCTAAAACAAAGTTAGGTGGCTCAATGAGCATCATTGC-3′) with the complementary 5′^32^P radiolabeled oligonucleotide AttI1′. The ds*att*C containing donor substrate (120 bp) was obtained by annealing the 5′^32^P radiolabeled oligonucleotide AttC2 (5′-CGCCCGTCTAACAATTCGTTCAAGCCGACGTTGCTTCGTGGCGGCGCTTGCGTGCTACGCTAAGCTTCGCACGCCGCTTGCCACTGCGCACCGCGGCTTAACTCAGGCGTTAGATGCACT-3′) with the complementary 5′^32^P radiolabeled oligonucleotide AttC2′. The receptor plasmids pGEM-T-*att*I1 (called p*att*I1) and pGEM-T-*att*C (called p*att*C) were previously described [11] and were purified under their closed circular forms using the Wizard SV gel excision kit (PROMEGA). The quality of the template was checked after purification by agarose gel stained with etidium bromide. Under these conditions about 90% of the detected DNA was found under closed circular structure and 10% under open circular form.

Generation of the internally radiolabeled single-stranded recombination fragment for DNA gel shift assays was performed by asymmetric PCR as previously reported ^11^. For this purpose, the primer of the desired strand was 10-fold more concentrated than the other. PCR was done in presence of [α−^32^P]dCTP. After amplification two products were separated on agarose gel: one of them showed the mobility of the double-stranded fragment and the other was identified as the ssDNA strand by its S1 nuclease susceptibility. The fragment was then eluted.

The recombination reaction was performed by incubation of the purified IntI1(his)_6_ (1 to 10 pmoles) with both donor and receptor substrates (0.1 to 0.2 pmoles) for 20 min at 4°C in a total volume of 5 µl to promote IntI1-DNA complexes. Then, the incubation proceeded at 37°C for 90 min in the presence of 7.5 mM Mg^++^, 50 mM TrisHCl pH 7.5 and 1 mM DTT in a total volume of 20 µl. Reaction fractions were treated by protease K (50 µg/ml) for one hour at 55°C and were then submitted to phenol/chloroform/isoamylalcohol (25/25/1, v/v/v) extraction. The aqueous fraction was loaded on vertical 1% agarose gel and run at 200 V. The gel was dried and autoradiographied.

### DNA binding assay

To determine its DNA binding activity, purified IntI1(his)_6_ (1 to 10 pmoles) was incubated either with the 5′ radiolabeled double-stranded or single-stranded *att*I1 fragment or with the 5′ radiolabeled double-stranded or single-stranded *att*C fragment for 20 min at 4°C in a total volume of 20 µl. The IntI1-DNA complexes were then loaded on vertical 1% agarose gel and run at 50 V for 4 hours at 4°C. The gel was then dried and autoradiographied. Quantification was performed by filter binding assays. Nitrocellulose filters (0.45 µm, Whatman) were treated with a solution of KOH 0.4 M and washed twice with water and 2 ml of pre-washing buffer (HEPES 20 mM; pH 7.5; MnCl_2_ 10 mM; NaCl 10 mM; calf thymus DNA 100 µg/ml). IntI1 was incubated under *in vitro* assay conditions for 20 min at 4°C with the different radiolabeled substrates (10 000 cpm, “A” value). After addition of 1 ml washing buffer (HEPES 20 mM; pH 7.5; MnCl_2_ 10 mM; NaCl 30 mM) to the reaction mix, the solution was filtered. Filters were washed twice with 4 ml of washing buffer. The radioactivity retained on filters was quantitated (“B” value) with a scintillation counter (Wallac 1409). In parallel a control was performed without protein to determine the negative background and about 1–10% of the radioactivity (“C” value) were retained on filters. The % of substrate bound to the protein was evaluated by calculating as follow: [(B−C)/A]×100.

Random ODN used in the preincubation experiments has the following sequence (100 bp) 5′-CTAGCTTAAGCTCGACTAGCTTGGTGCCCTAGCTAGCTTGAACCGATCTTACCTTGACGTCGGTAGGCGATGGCTAGCTTGGGTAGCTGTGTCATCGTAG-3′.

### Chemical crosslinking

Crosslinking experiments were performed as previously reported [17]. Highly purified IntI1 (2 pmoles) was incubated with radiolabeled ODNs (0.1 pmole) in the presence of 300 µM *cis*-aquahydroxydiamino-platinum (AHDAP), 0.05% NP40, 0.5 M Mg acetate, 10 mM DTT, 20 mM HEPES pH 7.5 at 37°C in the dark in a final volume of 10 µl. The platinum derivative was prepared as described before [19]. The reaction was stopped by eliminating the excess of AHDAP after crosslinking by elution through a G25 MicroSpin column (Amersham). Complexes were loaded on SDS-PAGE 12% after DNase I treatment and autoradiographied.
